# Manual reduction in testicular torsion and subsequent treatment after successful reduction: a series of reports in a single institution

**DOI:** 10.3389/fped.2024.1362104

**Published:** 2024-03-11

**Authors:** Xiaokang Qi, Junjie Yu, Xuefei Ding, Yehua Wang, Haiyan Zhu

**Affiliations:** ^1^ Department of Urology, Northern Jiangsu People’s Hospital, Clinical Medical College, Yangzhou University, Yangzhou, China; ^2^ Department of Day Surgery Ward, Northern Jiangsu People’s Hospital, Clinical Medical College, Yangzhou University, Yangzhou, China

**Keywords:** testicular torsion, manual reduction, bilateral orchiopexy, emergency treatment, conservative treatment

## Abstract

**Introduction:**

To explore the factors affecting the success of testicular torsion manual reduction and the safety of subsequent conservative treatment after successful reduction.

**Methods:**

Clinical data of 66 patients with testicular torsion treated in our emergency department from February 2017 to February 2022 were retrospectively collected. Manual reduction without anesthesia was performed in 19 patients. Patients with successful manual reduction chose different subsequent treatments according to the wishes of themselves and their guardians, including continuing conservative treatment and surgical exploration. Relevant clinical data were collected and analyzed.

**Results:**

Manual reduction was successful in 11 patients (11/19). Seven of them chose to continue conservative treatment, and four underwent surgical exploration immediately. Among the 7 patients who were treated conservatively, 3 underwent surgical treatment due to scrotal discomfort or testicular torsion at different stages, and the remaining 4 patients showed no recurrence of torsion during follow-up. Compared with other patients, patients with successful manual reduction had the shorter duration of pain (*p* < 0.05). The time from visiting our hospital to surgery in patients who attempted manual reduction was slightly shorter than those who underwent surgery directly (*p* > 0.05). The testes of these 11 patients were all successfully preserved.

**Conclusions:**

The short duration of pain may contribute to the success of manual reduction, and manual reduction did not increase the preparation time before surgery. Due to the unpredictable risk of recurrence, immediate surgical treatment is still recommended, or postponed elective surgical treatment should be offered in the next days or weeks.

## Introduction

Testicular torsion (TT) is one of the acute diseases requiring emergency treatment in urology, the annual incidence rate of TT in men under 18 years of age is estimated to be 3.8–5.9 per 100,000 (1, 2). TT is not common, but the rate of orchiectomy in surgical treatment is up to 33.6%−41.9% (1, 2). The survival of testicular tissue after TT depends on the degree and duration of TT (3), the duration of symptoms is the only predictor of successful testicular salvage after TT in children (4, 5). Testicular survival was 97.2% within 0–6 h after onset and decreased significantly after 12 h (6).

Although surgical treatment is the first option once TT is diagnosed, some scholars have suggested that manual reduction may be attempted, because manual reduction is simple and immediately available, which can improve the testicular preservation rate and may make surgery a non-emergency orchiopexy (7–10). A study with a median follow-up period of 21.5 months showed that manual reduction was safe and appeared to be part of treatment when applied together with elective orchiopexy (11). However, manual reduction of TT is not suitable for all patients. Doctors need certain handling skills and experience, and the selection of cases is also very important. Patient's pain, incomplete de-torsion, and the possibility of rotating the testicle in the wrong direction hampered the success of this technique (12). Possibly due to case selection bias, the success rate of manual reduction of TT varies significantly, ranging from 26% to 95.1% (7, 9–11). In previous reports of manual reduction, most of the patients were recommended and received surgical exploration (most orchiopexy and few orchiectomy) after successful reduction, while very few patients chose not to undergo surgery (7, 9–11).

In the past 5 years, 66 cases of TT were diagnosed and treated in emergency in our hospital, including 11 cases with successful manual reduction. These 11 patients chose to continue conservative treatment or surgical treatment. We collected and analyzed the clinical data of these patients to observe the factors affecting the success of TT manual reduction and the safety of subsequent conservative treatment after successful reduction.

## Methods

### Patients

Clinical data of patients with TT diagnosed in emergency of Northern Jiangsu People's Hospital from February 2017 to February 2022 were retrospectively collected. As the largest general hospital in a prefecture-level city, our hospital serves millions of people around. Patients with suspected TT for several days without emergency department visits and without emergency surgical treatment were excluded. The diagnosis was determined by clinical history, physical examination, and emergency ultrasound. Physical examination showed unilateral testicular tenderness, abnormal location (elevation and transverse) of the testicle, and scrotal swelling. Our emergency department has independent sonographer, ultrasound room and multiple ultrasound equipment, including a mobile ultrasound machine. For patients with suspected TT, emergency ultrasound can be performed immediately within a few minutes. The blood flow to the testicles was significantly reduced, or completely absent in ultrasound examination, a Whirlpool sign could be seen in some cases. The criteria for successful reduction of TT are complete relief of testicular pain and restoration of testicular blood supply as seen on ultrasound. The date of patients’ age, duration of pain, transferred from other hospitals or not, TT side, time from visiting our hospital to surgery, orchiectomy or orchiopexy in surgery and follow up were collected. These data were compared in patients with successful manual reduction, patients with failed reduction, and patients without manual reduction.

### Manual reduction procedure

All patients with confirmed TT are routinely told that surgery is required, and whether to perform manual reduction of TT depends on the urologist personal judgment. Manual reduction shall be carried out according to the following procedures: (a) Firstly, inform the patient and his parents that manual reduction can be attempted. During this period, there may be pain without anesthesia, and torsion may worsen or alleviate. Patients who refuse to attempt manual reduction directly enter the surgical exploration process. (b) After receiving their consent, let the patient stand, gently pull the testicle longitudinally, and release quickly. For those with mild swelling in a short time, the testicle may retract upward and rotate to the opposite direction of the torsion [similar to the principle of elastic retraction (9)]. (c) Ask the patient to lie flat on the bed and let him relax. Fix the upper skin of the scrotum with the left hand and gently hold the testicle with the right hand to reset it in the de-torsion direction. If the direction of TT cannot be determined by this method, due to 2/3 of testicles rotate in the inner direction (13) (counterclockwise in the left testicle and clockwise in the right testicle), try to rotate the testicle in the opposite direction for half or one turn first. If the pain is relieved and the position of the testicle is lowered, it indicates that the direction of the reduction is correct, or the reduction is successful. Continue to reduce 180°–360° or even more depending on testicular location and patient feedback on scrotal pain. If the operator feels significant resistance and the patient experiences increased pain, suggesting the testicular rotation was in the outer direction (counterclockwise in the right testicle and clockwise in the left testicle), try to rotate the testicle in the inner direction in the same way. (d) If testicular reduction is attempted in the second direction and the patient still has no tendency of pain relief, the de-torsion maneuver should be abandoned to avoid worsening TT. (e) If the patient’s pain is relieved immediately, the testicular position returns to normal and the testicular tension is reduced, an ultrasound should be performed to check the testicular blood supply. Real-time ultrasound monitoring of the testicular blood flow signal is useful when a movable ultrasound machine is available in the emergency treatment room at the time, so that examination and reduction can be carried out simultaneously. If the blood supply to the testicle is restored and the Whirlpool sign disappears, the manual reduction of TT can be considered successful.

Inform patients and parents that there may be residual spermatic cord distortion and TT may occur again, and strongly recommend further surgical exploration and testicular fixation. Patients who agreed to the surgery underwent emergency admission immediately, and surgical exploration would be performed as soon as possible. Patients who refused surgical treatment for the time being were recommended quiet rest and observation, and ultrasound should be performed again 6 h later to confirm testicular blood supply. And solemnly inform patients and parents of TT-related knowledge, if similar testicular pain occurs again, be sure to see a doctor in time. They are also advised to perform orchiopexy in the next days or weeks after important exams or during summer and winter vacations.

### Statistical analysis

The data were analyzed by SPSS 24.0 software. The count data were expressed as rate (%), and Chi-square test was used for comparison between groups. The measurement data were expressed as mean and standard deviation (mean ± SD), and the comparison between the groups was carried out by One-way analysis of variance, and *P*-value equal or less than 0.05 was considered to be statistically significant.

## Results

In this study, 66 patients with TT were enrolled, with a mean age of 14 ± 3.42 years. Forty-seven patients received surgical treatment directly, nineteen patients underwent manual reduction, and 11 of them were successful. There was no difference in age among the groups (Table 1). Patients with manual reduction had shorter pain duration than those with direct surgical treatment (*p *< 0.05). Patients with successful manual reduction had the shortest pain duration (mean 6.60 ± 13.80 h), but there was no significant difference compared with patients with failed manual reduction (mean 9.48 ± 6.63 h) (*p *= 0.912). There were no significant differences in referral rates, preoperative waiting time, and left/right torsion among the three groups (*p *> 0.05) (Table 1). Of the 11 patients with successful manual reduction, four underwent surgical treatment (Table 2). These four patients showed normal testicular position during surgery and successfully preserved the testicles. In patients with failed manual reduction and patients underwent surgical treatment directly, the proportion of orchidectomy was about 50%.

**Table 1:** Comparison of different treatment procedures for testicular torsion.

Characteristics	Manual reduction successful (*n* = 11)	Manual reduction failure (*n* = 8)	Non manual reduction (*n* = 47)	*P* value for variance analysis/*χ*^2^
Age (years)	14.82 ± 1.54	15.00 ± 2.88	14.94 ± 3.93	0.993
Duration of pain, hours (mean ± SD) (median, range)	6.60 ± 13.80^a^ 3, 1–48	9.48 ± 6.63^b^ 8, 3–24	30.37 ± 36.79^c^ 7, 1–168	0.040
Time from visiting our hospital to surgery, hours	3.25 ± 1.04*	3.11 ± 0.59	3.87 ± 1.50	0.292
Transferred from other hospitals, No. (%)	2 (18.2%))	1 (12.5%)	23 (48.9%)	0.061
Side, No. (left/right)	8 (72.7%)/3(27.3%)	4 (50.0%)/4 (50.0%)	24 (51.1%)/23 (48.9%)	0.464
Orchiectomy/orchiopexy, No.	0 (0%)/4 (100%)	4 (50%)/4 (50%)	23 (48.9%)/24 (51.1%)	0.176

a vs. b *p *= 0.912; a vs. c *p* = 0.003; b vs. c *p* = 0.002.

* *n* = 4.

**Table 2:** Clinical characteristics of 11 patients with successful manual reduction.

Patient	Age (years)	Side	Initial symptom		Duration of symptom (h)	Direction of torsion	Ultrasound doppler findings		Subsequent treatment	Pain again in follow-up (occurrence time)	Follow-up duration (month)
			Scrotal pain	Lower abdominal pain			Testicular blood flow	Whirlpool sign			
1	13	Left	Yes	–	1	Inner	Complete absent	–	CT	Yes (Two years)	66
2	14	Left	Yes	–	4	Inner	Complete absent	–	CT	Yes (Five days)	51
3	17	Left	Yes	–	48 (Worsened 2 h before hospital arrival)	Inner	Decreased blood	–	ST	No	44
4	14	Right	Yes	–	1	Outer	Complete absent	–	ST	No	40
5	15	Left	–	Yes	1	Outer	Complete absent	–	ST	No	37
6	16	Left	–	Yes	3	Inner	Decreased blood	Yes	CT	No	32
7	13	Left	Yes	–	3	Inner	Complete absent	Yes	CT	Yes (Two months)	24
8	16	Left	–	Yes (with inguinal mass)	5	Outer	Complete absent	–	CT	No	16
9	13	Right	Yes	Yes	2	Outer	Complete absent	Yes	CT	No	14
10	17	Left	Yes	–	3	Inner	Complete absent	–	CT	No	10
11	15	Right	Yes	–	1.5	Inner	Complete absent	–	ST	No	10

CT, conservative treatment, ST, surgical treatment.

**Figure 1:**
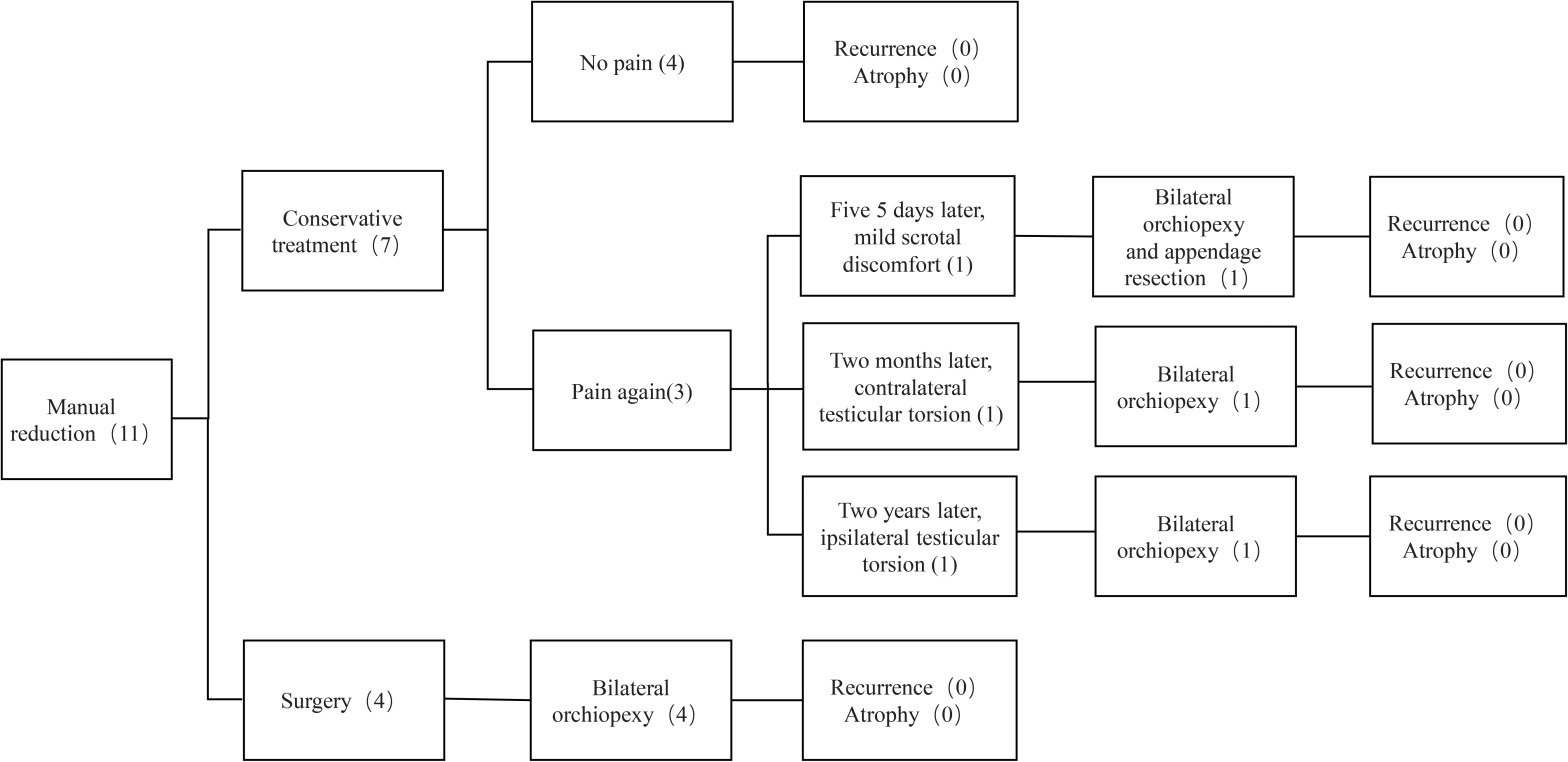
Treatment and follow-up of 11 patients with successful manual reduction.

Seven patients with successful manual reduction and their parents refused surgical treatment at the time due to the approaching academic test. Ultrasound re-examination of these seven patients 6 h later showed that the testicles were in good position and the blood supply was normal. Patient 2 had intermittent mild scrotal discomfort 5 days later. He was seen at another hospital and underwent exploratory surgery. However, the patient did not provide a paper discharge record from the external hospital, they told us that the testicular position was normal during the exploration in that hospital, and the doctors performed bilateral orchiopexy and resection of the testicular appendage. More precise information was not available through his parents. Another two conservatively treated patients developed contralateral and ipsilateral TT again 2 months and 2 years later, respectively. Due to good knowledge of TT, the patients realized that TT may have occurred again, and they all arrived at the hospital within 2 h. They were hospitalized for emergency scrotal exploration and bilateral orchiopexy on time. The remaining four conservatively treated patients and four surgically treated patients had no scrotal pain or TT during follow-up. (Figure 1). Testicles were preserved in all 11 patients with successful manual reduction. Their median follow-up time was 32 months (range, 10–66 months), and the testicular size did not decrease compared to their contralateral testicles.

## Discussion

TT can have adverse consequences, and the key to treatment is to restore blood supply to the testicle as soon as possible (5). Many factors can affect the prognosis of TT, such as late clinic visit, diagnostic delay or misdiagnosis, hospital transfer, preoperative examination, etc. (14–18). Once TT is diagnosed, it is universally accepted that emergency surgery should be performed immediately (12). The necessary preoperative examination and preparation can take more than 2–3 h (10, 19), and it is possible to restore testicular blood supply for the first time if manual reduction is attempted after the diagnosis. Demibras (11) tried manual reduction in 26 TT patients and achieved success in 20 patients, and 18 of them underwent sub-emergency surgery later (median 10 days, 0–45 days). Compared to those patients with emergency orchiopexy who did not undergo manual reduction, it can reduce the duration of pain and the time of testicular ischemia (11). In another study, significant differences in testicular salvage were found between patients who underwent or did not undergo manual reduction (7). 97.2% of the patients (70/72) with successful reduction received orchiopexy, while 75.4% (46/61) who did not attempt reduction or who had failed reduction received orchiopexy (7). Meanwhile, TT reduction immediately after diagnosis was superior to delayed reduction in testicular preservation, with corresponding testicular salvage rates of 93% (54/58) and 82% (33/40), respectively (17).

Although manual reduction of TT can achieve better results, not all patients are suitable for manual reduction. Even if manual reduction was successful, surgical exploration could find a small number of patients with residual cord torsion or incomplete reduction (13, 17). Patient discomfort, incomplete detorsion, and wrong rotating direction hamper the success of the success of manual reduction (12). The success rate of manual reduction of TT in previous reports varies greatly from 26% to 95.1% (7, 9–11), this may be due to the different rates of manual reduction attempted in all patients with TT and the inconsistent criteria in selecting patients for manual reduction, as well as bias in physician experience and practice. The average age of patients attempting TT reduction ranged from 10.8 to 20 years (7–11). In order to get reliable pain feedback from patients and better judge whether the reduction is successful, as well as to simplify the process and save time, manual reduction should be performed without anesthesia (9, 10). Manual reduction cannot be performed if the patient is too young to tolerate pain. Furthermore, prolonged torsion is often accompanied by obvious tissue edema or secondary hydrocele, which significantly increases the difficulty of manual reduction and is even not suitable for manual reduction (9, 20). In the previous literature, the average time from scrotal pain to hospital visit was 3–7.5 h in patients with TT who had successful manual reduction (7–11), and the effectiveness of manual reduction of TT depends on the duration of the ischemia period before manipulation (20). Furthermore, we found that attempting manual reduction did not increase the preoperative preparation time.

After successful manual reduction, most patients received surgical treatment, and very few patients chose conservative treatment (7, 9–11). There is little data on the probability of TT recurrence after successful manual reduction. In today's China, young students have heavy learning tasks and fierce competition for further education. Due to concerns about the impact of the operation on their studies and the trauma of the surgery, seven patients and their guardians chose to continue conservative treatment in this study. During follow-up, three of them did not avoid surgery due to scrotal discomfort or recurrent TT (affected or contralateral testicle), while the rest four patients did not experience scrotal pain during follow-up.

Although conservative treatment after successful manual reduction may be an option that can be considered in some patients, this choice should also be very cautious, unless the patient and their family make a firm decision and are aware of the risks involved. The authors believe that surgical exploration should be performed in the following cases which may be high-risk factors for recurrence: (a) Patients who still have scrotal pain or discomfort after manual reduction may have incomplete reduction or a torsion of the testicular appendage. The proportion of residual torsion after manual reduction can reach 29%–32% (13, 17). (b) The most common anatomical factor related to spermatic cord torsion has been reported to be high insertion of the tunica vaginalis (21). Patients with long and loose sperm cords have greater testicular mobility and are more likely to have recurrent TT. (c) Intermittent TT is characterized mainly by recurrent episodes of sudden unilateral scrotal pain that could resolve spontaneously. Although intermittent TT has a high rate of successful manual reduction and testicular salvage, surgical treatment should be performed to prevent testicular ischemic injury and interrupt repeated cycles of scrotal pain (22, 23). (d) Patients with a history of successful manual reduction of TT or surgery of TT before. (e) The patient has only one testicle left for various reasons. More reliable treatment must be taken to ensure the survival of the testicle.

Conservative treatment may not be smooth, and the relevant risks need to be informed in detail. It is necessary to repeat the ultrasound examination in a short time to determine the blood supply of the testicle. The most important thing is to educate patients and their families about TT and inform them they need to see a doctor in time if scrotal pain reoccurs. In this study, testicles were preserved in all 11 patients and testicular atrophy was not detected. This may be due to the small number of cases in this study and the short time from onset to hospital visit. Although patient 3 had a relatively long onset time, he has a history of recurring testicular pain similar to that on the affected side, he might have intermittent testicular torsion. No testicular atrophy was observed, probably because he was not in a state of continuous testicular torsion during these 2 days, or the torsion was not severe in the initial hours because the patient had an increase in pain 2 h before this presentation. Patient 2 underwent bilateral orchiopexy and testicular appendage resection at another hospital 5 days later. Based on the current information, we speculate that he may also have intermittent testicular torsion, and the torsion resolved spontaneously when he underwent surgery.

There are some limitations in our study. First, the successful manual reduction group had more advantageous factors in the data, there was no statistical difference except for the pain duration, which may be due to the relatively small number of cases. It is also possible that the success of TT reduction may have a certain chance, which should be studied more. Second, a small degree of torsion may also be another favorable factor for successful reduction, but the degree of torsion cannot be accurately determined like open surgery under direct vision. Therefore, no relevant comparison was made. Third, this is a retrospective study, and the case grouping may be affected by selection bias because 47 patients did not undergo manual reduction for various reasons.

Based on the limited clinical data above, it appears some contingency in the success of manual testicular reduction. However, manual reduction did not increase the preparation time before surgery, and short duration of pain may be a favorable factor for manual reduction. Although continued conservative treatment may be safe for individual patients after successful manual reduction, there are still some patients who cannot avoid surgery due to unpredictable recurrence, so immediate surgical treatment is still recommended, or postponed elective surgical treatment should be offered in the next days or weeks.

## Data Availability

The raw data supporting the conclusions of this article will be made available by the authors, without undue reservation.
